# NOX2-generated oxidative stress is associated with severity of ultrasound liver steatosis in patients with non-alcoholic fatty liver disease

**DOI:** 10.1186/1471-230X-14-81

**Published:** 2014-04-23

**Authors:** Maria Del Ben, Licia Polimeni, Roberto Carnevale, Simona Bartimoccia, Cristina Nocella, Francesco Baratta, Lorenzo Loffredo, Pasquale Pignatelli, Francesco Violi, Francesco Angelico

**Affiliations:** 1 Department of Internal Medicine and Medical Specialties, Sapienza University, Rome, Italy; 2 Department of Public Health and Infectious Disease, Sapienza University, Rome, Italy; 3 I Clinica Medica – Policlinico Umberto I, Viale del Policlinico 155, 00161 Rome, Italy

**Keywords:** Oxidative stress, Non-alcoholic fatty liver, 8-iso-PGF2α, sNOX2-dp, Metabolic syndrome

## Abstract

**Background:**

Chronic oxidative stress is one of the key mechanisms responsible for disease progression in non-alcoholic fatty liver disease. However, so far, few studies reported increased circulating levels of oxidative stress markers in patients with non-alcoholic fatty liver and no study has been performed with newer markers of systemic oxidative stress. The aim was to assess the relationship between urinary 8-iso-prostaglandin F2α and serum soluble NOX2-derived peptide and the severity of liver steatosis in subjects with non-alcoholic fatty liver.

**Methods:**

The study was performed in 264 consecutive patients referred for suspected metabolic disease. Steatosis was defined according to Hamaguchi ultrasonographic criteria. Oxidative stress was assessed by urinary 8-iso- prostaglandin F2α and serum soluble NOX2-derived peptide levels.

**Results:**

Patients with non-alcoholic fatty liver had higher (p < 0.001) mean values of urinary 8-iso-PGF2α and of serum soluble NOX2-derived peptide, alanine aminotransferase, Cytokeratin-18 and homeostasis model of insulin resistance and lower values of serum adiponectin as compared to those without. Prevalence of metabolic syndrome and of its clinical features was significantly higher in patients with non-alcoholic fatty liver. Same findings were also observed after the exclusion of obese subjects, or subjects with diabetes or with metabolic syndrome and in those not taking statin medication. In addition, the levels of urinary 8-iso-PGF2α were independent predictors of non-alcoholic fatty liver and a strong association of urinary 8-iso-PGF2α and of serum soluble NOX2-derived peptide with the severity of steatosis at ultrasound was also observed.

**Conclusions:**

We demonstrated increased markers of oxidative stress in subjects with non-alcoholic fatty liver. Urinary 8-iso-PGF2α and serum soluble NOX2-derived peptide levels were independent from obesity, diabetes and metabolic syndrome and increased with the severity of liver steatosis at ultrasound.

## Background

Nonalcoholic fatty liver disease (NAFLD) includes a wide spectrum of liver diseases ranging from simple fatty liver to non-alcoholic steatohepatitis (NASH), which may progress to fibrosis and even cirrhosis and hepatocellular carcinoma [1]. It represents the most common and emerging chronic liver disease worldwide [2]. NAFLD is strongly associated with obesity, insulin resistance, hypertension, and dyslipidemia, and is now regarded as the liver manifestation of the metabolic syndrome (MetS). a cluster of metabolic and cardiovascular risk factors including systemic inflammation and oxidative stress [3-8].

Traditionally, according to the “two hit” theory, simple steatosis and NASH have been considered an histological continuum with increasing degrees of severity [9]. More recently, the ‘multiple parallel hits’ hypothesis’ has been proposed, suggesting simple steatosis and NASH as two unrelated disorders [10,11].

Several lines of evidence suggest that chronic oxidative stress is one of the key mechanisms responsible for liver damage and disease progression in NAFLD [9]. In particular, according to the “two-hit” theory, oxidative stress is a major player triggering the progression of steatosis to NASH as the result of an imbalance between pro-oxidant and anti-oxidant chemicals that lead to liver cell damage. In fact, the increased production of reactive oxygen species (ROS) is known to cause lipid peroxidation, followed by inflammation, and activation of stellate cells leading to fibrogenesis. Therefore, although the mechanisms underlying disease progression remain poorly understood, a therapeutic strategy targeting oxidative stress reduction has been proposed and, based on the results of a single randomized controlled trial [12], supplementation with vitamin E has been suggested by recent AASLD guidelines for the treatment of NASH in non diabetic subjects [13].

So far, few studies reported increased circulating levels of oxidative stress markers in patients with NAFLD [14-19]. However, most studies contained small number of patients and no study was able to document the relationship between the extent of steatosis and systemic markers of oxidative stress [20]. Larger studies with newer markers of oxidative stress are required since routine blood oxidative stress tests are unreliable markers of hepatic steatosis and probably do not accurately reflect hepatic oxidative stress. In this study, to assess oxidative stress in vivo, we measured urinary 8-iso-prostaglandin F2α (8-iso-PGF2α) and serum levels of soluble NOX2-derived peptide (sNOX2-dp). Measurement of urinary 8-iso-PGF2α is widely accepted as reliable indicator of oxidative stress in vivo [21,22]. Soluble NOX2-dp is a marker of NOX2 activation by blood cells, which is a member of the NADPH oxidase family which plays an important role in ROS generation [23,24]. Elevated urinary 8-iso-PGF2α and serum sNOX2-dp levels have been described in a number of chronic inflammatory and metabolic diseases [25-28].

Aim of the present study was to assess the relationship between urinary 8-iso-PGF2α and serum sNOX2-dp and the severity of liver steatosis in subjects with NAFLD in different clinical settings.

## Methods

### Study patients

The study has been performed in 264 consecutive patients referred to our metabolic outpatient clinic for suspected metabolic disease, who had a liver ultrasonographic scanning (US) performed as part of routine clinical examination. To be eligible for the study, patients had to have fulfilled the following criteria: no history of current or past excessive alcohol drinking as defined by an average daily consumption of alcohol >20 g; negative tests for the presence of hepatitis B surface antigen and antibody to hepatitis C virus; absence of history and clinical, biochemical and US findings consistent with cirrhosis and other chronic liver diseases. None of the subjects were taking amiodarone and other drugs known to promote fatty liver disease. Subjects underwent routine clinical and biochemical evaluation. Waist circumference, height and weight were recorded and body mass index (BMI) was calculated as weight (Kg) divided by height (m^2^). Blood pressure was recorded following standard procedures. Diabetes was diagnosed according to the WHO criteria [29]. Subjects taking insulin or oral antidiabetic drugs were considered to have diabetes. According to the modified criteria of the ATP III Expert Panel of the US National Cholesterol Education Program [30], MetS was diagnosed on the concomitant presence of at least three of the following five clinical features: waist circumference (central obesity) > 102 cm in men and > 88 cm in women, fasting blood glucose ≥ 100 mg/dl, triglycerides ≥ 150 mg/dl, HDL-cholesterol < 40 mg/dl in men and < 50 mg/dl in women, arterial systolic/diastolic blood pressure ≥130/≥ 85 mm/Hg. A metabolic score was calculated for each patient based on the number of the discrete components of MetS identified. Written informed consent was obtained from all patients before the study. The study was approved by the ethics committee of the Policlinico Umberto 1 Hospital of Rome and conforms to the ethical guidelines of the 1975 Declaration of Helsinki.

### Laboratory measurements

A venous blood sample and a spot urine sample were collected after a 10-14-h overnight fast. Serum total cholesterol, HDL-cholesterol and triglycerides were measured by an Olympus AN 560 apparatus using an enzymatic colorimetric method. LDL-cholesterol levels were calculated according to the Friedwald formula. Plasma insulin levels were assayed by commercially available radioimmunoassay. The homeostasis model of insulin resistance (HOMA-IR), based on serum fasting glucose and insulin levels, was used as a measure of insulin resistance [31]. Urinary 8-iso-prostaglandin F2α (8-iso-PGF2α), as marker of whole body oxidative stress, was measured by a previously described and validated enzyme immunoassay method [32]. Intra-assay and inter-assay coefficients of variation were 2.1% and 4.5%, respectively. Serum levels of soluble NOX2-derived peptide (sNOX2-dp) were detected by ELISA method as previously described [33]; intra-assay and inter-assay coefficients of variation were 5.2% and 6%, respectively. Values are expressed as pg/ml. Adiponectin (APN) serum levels were measured with a commercial immunoassay (TemaRicerca, Italy). Intra-assay and inter-assay coefficients of variation were 6 and 8%, respectively. Serum levels of Cytokeratin 18-M30 (CK-18) were measured as marker of liver damage with a commercial immunoassay (Tema Ricerca, Italy) and expressed as mlU/ml. Intra-assay and inter-assay coefficients were 6% and 7% respectively.

### Ultrasonographic examination

Liver US scanning was performed to assess the degree of steatosis. All US were performed by the same operator who was blinded to laboratory values using an Esaote Medica apparatus equipped with a convex 3,5 MHz probe. Liver steatosis was defined according to Hamaguchi criteria based on the presence of abnormally intense, high level echoes arising from the hepatic parenchyma, liver-kidney difference in echo amplitude, echo penetration into deep portion of the liver and clarity of liver blood vessel structure [34]. Steatosis was assessed semi-quantitatively on a scale of 0–6: 0, absent; 1,2 mild; 3,4 moderate; 5,6 severe.

### Statistical analysis

Statistical analysis was performed by using the SPSS statistical software version 8.0 for Windows (SPSS, Inc., Chicago. Illinois). Student’s t-test for unpaired data was used for the comparison of mean values. Distribution of continuous variables was tested for normality using the a Kolmogorov-Smirnov test. Data are expressed as the mean ± SD for normally distributed variables and as median followed by 25^th^ and 75^th^ centiles for non-normally distributed data. Group comparisons for normally distributed variables was performed by use of analysis of variance (ANOVA) and unpaired Student’s t-test when appropriate, Non normally distributed variables were tested by Mann–Whitney test and Kruskall-Wallis test. Proportions and categorical variables were tested by the χ^2^ –test and by the 2-tailed Fisher’s exact method when appropriate. All *P* values are two-tailed; a *P* value of less than 0.05 was considered to indicate statistical significance. Multiple linear regression analyses and a stepwise logistic regression analysis testing for the dichotomous response variable presence or absence of NAFLD were performed after controlling for possible clinical and biochemical confounders. The predictor variables entered in the different regression models were age, gender, BMI, diabetes, MetS, HOMA-IR, serum triglycerides, adiponectin, cytokeratin-18, urinary 8-iso-PGF2α and statin use.

## Results

Table 1 reports some clinical and biochemical characteristics of subjects with and without NAFLD. Patients with NAFLD had significantly higher (p < 0001) mean values of urinary 8-iso-PGF2α and of serum sNOX2-dp, ALT, CK-18 and HOMA-IR and lower values of serum adiponectin. Prevalence of MetS and of most of its clinical features was significantly higher in patients with NAFLD.

**Table 1:** Clinical and biochemical characteristics of subjects with and without NAFLD

	**NAFLD (213)**	**w/o NAFLD (51)**	**p**
Age (yrs)	54,3 ± 12	56,1 ± 14,4	Ns
Male Gender (%)	64,6	63,3	Ns
BMI (kg/m^2^)	31,6 ± 5,6	26,8 ± 3,6	<0,001
Urinary 8-iso-PGF2α (pg/mg creatinine)	714,4 ± 121,5	621,2 ± 125,9	<0,001
sNOX2-dp (pg/ml)	57,4 ± 13.6	47,8 ± 9.9	<0.001
Adiponectin (ng/ml)	8,5 (5/12)	13 (8/15)	<0,001
Cytokeratin 18 (mIU/ml)	180 (146/190)	136 (125/173)	<0,001
ALT (IU/L)	27,5 (20/40)	18 (14/27)	<0,001
HOMA_IR	3,5 (2,4/5,9)	1,9 (1,3/2,3)	<0,001
Metabolic syndrome (%)*	67,7	21,7	<0,001
High fasting glucose (%)*	53,8	29,8	< 0,01
Hypertriglyceridemia (%)*	46,9	14,6	<0,001
High waist circumference (%)*	81,9	42,6	<0,001
Low HDL-cholesterol (%)*	37,2	18,8	< 0,05
High blood pressure (%)*	84,5	75,5	Ns
Diabetes (%)	31,1	12,2	< 0,01
Statin use (%)	33,5	42,9	Ns

*According to ATPIII modified criteria (Ref. 30).

Bivariate correlation coefficients between the study variables are reported in Table 2. A strong positive correlation was found between Urinary 8-iso-PGF2α and serum sNOX2-dp (r = 0.745; p < 0.001). Both variables were positively correlated (p < 0.001) with Hamaguchi and MetS scores, BMI, serum CK-18 and HOMA-IR and negatively correlated (p < 0.001) with serum adiponectin.

**Table 2:** Linear correlation coefficients between some clinical and biochemical variables

	**PGF2α**								
Urinary 8-iso-PGF2α (pg/mg creatinine)	1	BMI							
Body mass index (kg/m2)	,803**	1	Age						
Age (yrs)	-,051	-,089	1	Adipo					
Adiponectin (ng/mL)	-,686**	-,838**	,004	1	CK-18				
Cytokeratin-18 (mIU/ml)	,684**	,723**	,011	-,602**	1	NOX2			
sNOX2-dp (pg/ml)	,745**	,570**	-,074	-,430**	,491**	1	ALT		
ALT (IU/L)	,002	,017	-,098	,031	,061	-,007	1	HOMA-IR	
HOMA-IR	,321**	,417**	,063	-299**	,354**	,288**	,254**	1	MetS code
MetS code§	,390**	,468**	,191**	-,405**	,436**	,351**	,143*	,536**	1
Hamaguchi score^	,374**	,477**	,012	-,369**	,339**	,325**	,416**	,497**	,481**

*p < .01; **p < .001; ^§^no. of variables of MetS according to ATPIII modified criteria (Ref. 30); ^see Methods.

In order to better evaluate the independent effect of NAFLD on the above variables, separate comparisons between subjects with and without NAFLD were performed in subgroups of subjects.

without diabetes, MetS, obesity and statin use (Table 3). In all subgroups, mean urinary 8-iso-PGF2α and serum sNOX2-dp were significantly higher in subjects with NAFLD, as compared with those without NAFLD. Moreover, most of the other differences were still statistically significant when comparisons were performed in subjects without diabetes, without obesity, without MetS and in those not taking statins. Prevalence of NAFLD and of severe steatosis significantly increased in the increasing tertiles of urinary 8-iso-PGF2α (66.3 vs 83.7 vs 91.9; p < 0.001 and 13.8 vs 30.3 vs 45.6: p < 0.001, respectively).

**Table 3:** Clinical and biochemical characteristics in subjects without diabetes, or obesity or metabolic syndrome and in statin non users according to the presence or absence of NAFLD

	**Subjects w/o diabetes**		**Subjects w/o obesity^**		**Subjects w/o MetS**		**Subjects w/o statin use**	
	**Nafld (144)**	**no-Nafld (43)**	**Nafld (85)**	**no-Nafld (39)**	**Nafld (73)**	**no-Nafld (41)**	**Nafld (139)**	**no-Nafld (28)**
Age (yrs)	52,1 ± 12,7	54,4 ± 14	55,0 ± 13,0	55,0 ± 14,7	50,1 ± 13,1	54,7 ± 13,0	50,8 ± 11,8	53,0 ± 15,9
Male gender (%)	33,3	34,9	23,8	34,2	31,8	30,6	36,0	39,3
BMI	30,9 ± 5,6	26,9 ± 3,7 ***	26,6 ± 2,3	25,2 ± 2,0***	29,5 ± 5,7	26,5 ± 3,5**	31,0 ± 5,8	27,2 ± 3,9***
Urinary 8-iso-PGF2α (pg/mg creatinine)	703,4 ± 117,9	634,1 ± 106,7***	626,6 ± 81,5	583,6 ± 102,7*	672,1 ± 123,0	619,3 ± 95,1*	711,6 ± 133	627,5 ± 147,9**
sNOX2-dp (pg/ml)	56,4 ± 13,8	49,9 ± 8,9***	49,4 ± 12,9	45,8 ± 8,9	54,0 ± 13,3	48,4 ± 8,3*	57,5 ± 14,1	47,5 ± 10,7***
Adiponectin (ng/ml)	8,5 (5,5/12)	13,5 (7,5/15)***	12,5 (10,5/14)	13,5 (12,5/14,5)**	10,5 (6,2/13,6)	13,0 (6,5/14)*	7,5 (5/11,5)	13,5 (6,8/15)***
Cytokeratin-18 (mIU/ml)	174,5 (136/189)	130,0 (125/175)**	149,0 (125/179)	126,0 (110/165)*	161,5 (125/180	165,0 (125/178)	179,0 (145/190)	140,0 (125/179)**
ALT (IU/L)	26 (20/40)	20 (15/27)***	34 (23/42)	15 (13/24)***	26 (19/27)	18 (14/27)**	27 (20/40)	18 (14/27)***
GGT (IU/L)	25 (17/40)*	20 (14/28)	30 (20/52)	21 (16/23)**	23 (16/10)	21 (18/36)	25 (17/44)*	21 (13/30)
HOMA_IR	2,9 (2,1/4,5)	1,8 (1,2/2,1)***	2,8 (1,8/4,3)	2,0 (1,6/3,2)	2,6 (1,7/3,4)	1,8 (1,5/2,1)*	3,1 (2,2/5,5)	1,8 (1,1/2,1)***
MetS^§^ (%)	57,0	12,5***	51,8	19,4***	0	0	57,8	16,0***
High Fasting Glucose^§^ (%)	32,9	19,5	44,0	30,6	15,2	13,9	41,3	23,1
High Triglycerides^§^ (%)	44,8	11,9***	39,3	16,2*	7,6	13,9	40,9	11,1**
High Waist Circumference^§^ (%)	78,2	43,9***	55,4	27,0**	59,1	38,9	80	46,2***
Low C-HDL^§^ (%)	35,7	16,7*	31,0	21,6	6,1	13,9	37,2	14,8*
High Blood Pressure^§^ (%)	79,0	74,4	81,0	73,7	69,7	75,0	80,3	75,0
Diabetes (%)	0	0	21,4	15,8	7,6	2,8	20,1	7,1
Statin use (%)	22,9	39,5*	31,0	47,4	13,6	41,7**	0	0

*p< ,05; **p< ,01; ***p< ,001; ^§^According to ATPIII modified criteria (Ref. 30); ^^^BMI > 30.0.

Table 4 reports mean values of some biochemical variables according to NAFLD severity at ultrasound examination. A progressive, statistically significant increase (p < .001) of mean values of urinary 8-iso-PGF2α and of serum sNOX2-dp, CK-18, ALT, gamma-glutamil-transpeptidase (GGT) and HOMA-IR was observed from the group without fatty liver to the groups with mild, moderate and severe steatosis, while a decreasing trend was observed for serum adiponectin.

**Table 4:** Some biochemical variables in all subjects and in subjects with MetS according to NAFLD severity at ultrasound examination

	**ALL SUBJECTS**					**SUBJECTS WITH MetS**				
**Variables**	**N A F L D**									
	**Absent (n = 51)**	**Mild (n = 45)**	**Moderate (n = 88)**	**Severe (n = 80)**	**P**	**Absent (n = 10)**	**Mild (n = 21)**	**Moderate (n = 54)**	**Severe (n = 63)**	**P**
Urinary 8-iso-PGF2α (pg/mg creatinine)	621,2 ± 125,9	674,3 ± 121,2	700,7 ± 108,4	751,3 ± 121,0	<,001	616,6 ± 213,2	700,2 ± 103,9	734,3 ± 34,5	761,9 ± 96,5	<,001
sNOX2-dp (pg/ml)	47,9 ± 9,9	52,9 ± 15,5	57,1 ± 13.3	60,0 ± 12,2	<.001	45,3 ± 14,2	56,0 ± 13,3	58,9 ± 12,6	60,4 ± 10.6	<,005
Adiponectin (ng/ml)	13,0 (8,0/15,0)	10,0 (5,5/12,0)	8,8 (6,0/13,5)	6,0 (4,9/9,0)	<,001	12,5 (5,0/13,9)	9,4 (5,8/11,8)	7,5 (5,0/11,1)	6,0 (4,6/8,5)	<,05
Cytokeratin 18 (mIU/ml)	136 (125/173)	169 (129/185)	176 (140/190)	180 (168/182)	<,001	147 (128/182)	170 (132/188)	180 (162/190)	182 (175/195)	<,05
ALT (IU/L)	18 (14/27)	21 (17/34)	26 (19/36)	34 (25/45)	<,001	16 (12,/22)	26 (19/37)	25,5 (17/36,3)	32 (24/43)	<,001
GGT (IU/L)	21 (14/30,5)	23 (16/40,7)	21 (16/35)	33,50 (24/61)	<,001	14 (11/25)	25 (19/42)	22 (16/34)	34 (24/64)	<,001
HOMA_IR	1,9 (1,3/2,3)	2,5 (1,7/3,8)	3,3 (2,2/5,4)	5,2 (3,1/7,3)	<,001	2,9 (1,1/3,8)	2,9 (1,7/4,5)	3,9 (2,9/5,9)	5,6 (3,3/7,4)	<,001
Metabolic syndrome (%)*	21,7	50,0	63,5	81,8	<,001	100	100	100	100	-

*Defined according to ATPIII modified criteria (Ref. 30).

Since urinary 8-iso-PGF2α and serum sNOX2-dp values were also significantly correlated with HOMA-IR and MetS score, a separate analysis was performed in patients with MetS. Also in this clinical setting, an increasing trend of the mean levels of both urinary 8-iso-PGF2α and serum sNOX2-dp with increasing NAFLD severity was observed.

Table 5 reports the results of the stepwise multiple logistic regression analysis performed to assess the independent contribution of urinary 8-iso-PGF2α for the prediction of NAFLD. Age, urinary 8-iso-PGF2α and MetS were independent predictors of NAFLD, after controlling for gender, BMI, HOMA-IR, serum triglycerides and adiponectin, diabetes and statin use.

**Table 5:** Stepwise multiple logistic analysis of independent predictors of NAFLD in 264 subjects

	**B**	**S.E.**	**P**	**O.R.**	**95,0% C.I. for O.R.**	
					**Lower**	**Upper**
Age (yrs)	-,048	,021	,021	,953	,915	,993
Urinary 8-isoprostanes (pg/mg creatinine)	,006	,002	,003	1,006	1,002	1,010
Metabolic syndrome	1,533	,535	,004	4,634	1,622	13,236

Variables entered on step 1: age, gender, BMI, *HOMA-IR*, serum triglycerides and adiponectin, urinary 8-isoprostanes, metabolic syndrome, diabetes, statin use.

Two more regression analyses were performed to evaluate factors independently related to the severity of the NAFLD (serum cytokeratin-18 levels) and factors independently related to the degree of systemic oxidative stress (urinary 8-iso-PGF2α).

In the first multiple linear regression analysis, BMI (standardized coefficient β = 0. 236; SE = 0.725; p = 0.019), urinary 8-iso-PGF2α (standardized coefficient β = 0.268; SE = 0.029; p = 0.002) and serum adiponectin (standardized coefficient β = −0.193; SE = 0.726; p = 0.012) were independent predictors of serum CK-18 levels (R^2^ = 0.39). In the second regression analysis, BMI (standardized coefficient β = 0.687; SE = 1.430; p = 0.000) and serum cytokeratin-18 (standardized coefficient β = 2.904; SE = 0.144; p = 0.004) were independently associated with urinary 8-iso-PGF2α levels (R^2^ = 0.66).

## Discussion

Our findings show, for the first time, an increased systemic oxidative stress in subjects with NAFLD, as assessed by increased levels of urinary 8-iso-PGF2α, currently regarded as the best measure of oxidative stress in vivo and of serum sNOX2-dp, a marker of NOX2 activation by blood cells, which plays an important role in ROS production [21-23].

So far, few studies have investigated markers of systemic oxidative stress in subjects with NAFLD. However, no study has documented the relationship between urinary 8-iso PGF2α and serum sNOX2-dp and the extent of fatty liver. In a small study performed in 21 subjects with NASH and 19 controls, subjects with NASH had significantly higher levels of oxidized LDL and of thiobarbituric acid-reacting substances (TBARS) suggesting an increased cardiovascular risk [14]. Similar results were reported in India, where TBARS levels were significantly elevated and GSH/GSSG ratio was significantly decreased in NAFLD subjects without and with type 2 diabetes [14]. Increased systemic levels of malondialdehyde were observed in 58 male patients with histologically proven NAFLD compared to healthy age matched males [15]. In a further study, NAFLD children with immune responses against MDA derived antigens showed more severe lobular inflammation and had a 13-fold higher prevalence of overt NASH suggesting the presence of oxidative stress in a high proportion of NAFLD children [17]. In two more study the percentage of hepatocytes positive for 8-OHdG expression and serum 8-OHdG levels were significantly higher in patients with NASH than simple fatty liver, while the oxidative stress marker GGT was increased in both conditions [18,35,36]. Finally, in a recent cross-sectional study, oxidative stress detected as the ratio of plasma total antioxidant status to total oxidant status was associated with insulin resistance in obese adolescents with NAFLD [19].

Our study was carried out in a large sample of consecutive patients referred for suspected metabolic disease, who had a liver US performed as part of routine clinical examination. We found statistically significant higher urinary 8-iso-PGF2α and serum sNOX2-dp levels in subjects with fatty liver, as compared to those without. The same findings were also observed after the exclusion of obese subjects, or subjects with diabetes or with MetS and in those not taking statin medication. In addition, the levels of urinary 8-iso-PGF2α were independent predictors of NAFLD and a strong association of urinary 8-iso-PGF2α and of serum sNOX2-dp with the severity of liver steatosis at ultrasound examination was also observed. In our study, a correlation was also found between HOMA-IR, urinary 8-iso PGF2α and sNOX2-dp, confirming the interdependency of insulin resistance and oxidative stress in the pathogenesis of NAFLD. Moreover, urinary 8-iso-PGF2α were also independent predictors of serum CK-18, a marker of apoptosis reflecting liver disease severity, further suggesting a possible role of oxidative stress in the progression from simple fatty liver to NASH [37].

Our results support the hypothesis that NAFLD is associated to enhanced oxidative stress as the result of increased 8-isoprostane production induced by NOX2 activation. Although the association between NAFLD and markers of increased oxidative stress does not necessary imply causality, our findings suggest oxidative stress as a possible target of antioxidant therapy in patients with NAFLD and give support to the indication for vitamin E supplementation for non diabetic, non cirrhotic patients with NASH [12,13].

Measurement of 8-iso-PGF2α has overcome many of the limitations associated with other methods and has emerged as the most reliable approach to assess the role of in vivo oxidative stress status in the pathogenesis of human disease. Isoprostanes are stable prostaglandin-like compounds that are produced by free radical mediated lipid peroxidation in vivo are as a result of oxidative damage to cell membranes. They are unaffected by dietary lipid composition and are increased in animal models of oxidant injury. Based on these characteristics, F2-isoprostanes have emerged as the gold standard of oxidative stress in vivo, especially if determined in urine [22,23]. In fact, measurement of urinary 8-iso-PGF2α has several advantages such as non invasiveness, stability and absence of any significant daily and day-to-day variability. We have previously demonstrated increased urinary 8-iso PGF2α values also in other chronic conditions such as hypercholesterolemia, metabolic syndrome, obstructive sleep apnoea syndrome and peripheral artery disease [25-28]. Recently, women with high levels of urinary 8-iso PGF2α were reported to have an 80% increased risk of dying of coronary heart disease or stroke, supporting the involvement of oxidative stress also in the pathophysiology of cardiovascular disease [36]. In our study, cytokeratin-18, a marker of liver disease severity, was an independent predictor of urinary 8-iso PGF2α, thus suggesting also a possible effect of fatty liver on systemic oxidative stress.

Our study may have some limitations. First, we detected fatty liver by ultrasound, which is a qualitative method inadequate to quantify less than 20% liver fat content [38]. Moreover, the Hamaguchi ultrasonographic score does not specifically predict NASH. The gold standard for the diagnosis of NASH is liver biopsy, but this is an invasive procedure with potentially serious complications and is therefore not acceptable without clinical indication. We acknowledge that grades of steatosis could have been better determined by magnetic resonance spectroscopy. However, Hamaguchi score showed 100% specificity and 91.7% sensitivity when compared with liver biopsy in NAFLD patients [34]. Second, although performed in a large series of patients, the study has been carried out in patients recruited in a Hospital-based setting. Finally, this is a cross-sectional study and it is therefore impossible to say whether oxidative stress, caused by obesity, contributes to the pathophysiology of NAFLD (or perhaps more NASH) or whether the inflamed liver contributes to the systemic oxidative stress.

## Conclusion

We demonstrated increased markers of oxidative stress in subjects with NAFLD. Urinary 8-iso-PGF2α and serum soluble NOX2-derived peptide levels were independent from obesity, diabetes and MetS and increased with the severity of liver steatosis at ultrasound. Our findings are consistent with the “two-hit” theory based on the prominent role of oxidative stress as a major player triggering the progression of steatosis to NASH. Moreover, they are also in agreement with the more recent “multiple parallel hits” theory including genetic predisposition, intestinal macrobiota and insulin resistance as the major contributors of increased oxidative stress and progressive liver damage [10]. Finally, they may also give support to the recommendation for early antioxidant treatment extended even to subjects with simple steatosis.

## Abbreviations

NAFLD: Non-alcoholic fatty liver disease; NASH: Non-alcoholic steatohepatitis; 8-iso-PGF2α: 8-iso-prostaglandin F2α; sNOX2-dp: Soluble NOX2-derived peptide; ALT: Alanine aminotransferase; CK-18: Cytokeratin-18; HOMA-IR: Homeostasis model of insulin resistance; MetS: Metabolic syndrome; ROS: Reactive oxygen species; US: Ultrasonographic scanning; BMI: Body mass index; TBARS: Thiobarbituric acid-reacting substances; GGT: Gamma-glutamil-transpeptidase.

## Competing interests

All authors declare that they have no competing interests.

## Authors’ contributions

MDB contributed to study design and wrote the manuscript. LP, RC, FB, SB and CN contributed to data collection, analysis and interpretation; LL and PP reviewed the manuscript; FV reviewed and edited the manuscript. FA designed the study and wrote the manuscript; he is the guarantor of this work and, as such, had full access to all the data in the study and takes responsibility for the integrity of the data and the accuracy of the data analysis. All Authors approved the final manuscript.

## Pre-publication history

The pre-publication history for this paper can be accessed here:

http://www.biomedcentral.com/1471-230X/14/81/prepub
